# Scoring System for Predicting the Risk of Liver Cancer among Diabetes Patients: A Random Survival Forest-Guided Approach

**DOI:** 10.3390/cancers16132310

**Published:** 2024-06-24

**Authors:** Sarah Tsz-Yui Yau, Eman Yee-Man Leung, Chi-Tim Hung, Martin Chi-Sang Wong, Ka-Chun Chong, Albert Lee, Eng-Kiong Yeoh

**Affiliations:** JC School of Public Health and Primary Care, The Chinese University of Hong Kong, Hong Kong, China; 1155075647@link.cuhk.edu.hk (S.T.-Y.Y.); cthung@cuhk.edu.hk (C.-T.H.); wong_martin@cuhk.edu.hk (M.C.-S.W.); marc@cuhk.edu.hk (K.-C.C.); alee@cuhk.edu.hk (A.L.)

**Keywords:** liver cancer, diabetes, risk prediction, survival data, random forest

## Abstract

**Simple Summary:**

Patients with diabetes are at greater risk of developing liver cancer than the general population. However, risk prediction models or scoring systems for liver cancer mostly focus on patients with preexisting liver diseases, and models for patients with diabetes or those in the absence of liver diseases remain rare. This study aims to develop a risk scoring system among diabetes patients and a sub-model for diabetes patients without cirrhosis or chronic viral hepatitis. In the final model, presence of chronic hepatitis B/C, cirrhosis, alanine aminotransferase, age, and sex were included as predictors. In the sub-model, alanine aminotransferase, triglycerides, age, and sex were included as predictors. Findings of the study may potentially provide individualized risk prediction for diabetes patients.

**Abstract:**

Background: Most liver cancer scoring systems focus on patients with preexisting liver diseases such as chronic viral hepatitis or liver cirrhosis. Patients with diabetes are at higher risk of developing liver cancer than the general population. However, liver cancer scoring systems for patients in the absence of liver diseases or those with diabetes remain rare. This study aims to develop a risk scoring system for liver cancer prediction among diabetes patients and a sub-model among diabetes patients without cirrhosis/chronic viral hepatitis. Methods: A retrospective cohort study was performed using electronic health records of Hong Kong. Patients who received diabetes care in general outpatient clinics between 2010 and 2019 without cancer history were included and followed up until December 2019. The outcome was diagnosis of liver cancer during follow-up. A risk scoring system was developed by applying random survival forest in variable selection, and Cox regression in weight assignment. Results: The liver cancer incidence was 0.92 per 1000 person-years. Patients who developed liver cancer (*n* = 1995) and those who remained free of cancer (*n* = 1969) during follow-up (median: 6.2 years) were selected for model building. In the final time-to-event scoring system, presence of chronic hepatitis B/C, alanine aminotransferase, age, presence of cirrhosis, and sex were included as predictors. The concordance index was 0.706 (95%CI: 0.676–0.741). In the sub-model for patients without cirrhosis/chronic viral hepatitis, alanine aminotransferase, age, triglycerides, and sex were selected as predictors. Conclusions: The proposed scoring system may provide a parsimonious score for liver cancer risk prediction among diabetes patients.

## 1. Introduction

Globally, liver cancer is the third leading cause of cancer death, accounting for approximately 800,000 deaths each year [1]. Major risk factors for liver cancer include chronic infection with hepatitis B/C virus, liver cirrhosis, and heavy alcohol use [2]. Other risk factors include metabolic conditions (obesity [3] and diabetes [4]) and tobacco use [5].

Various scoring systems have been developed for liver cancer prediction and risk stratification [6]. Variables commonly included in the existing scoring systems [6,7] are variables related to the liver or known etiologies of liver cancer, such as chronic hepatitis B/C status, cirrhosis, liver function (albumin, bilirubin, alanine aminotransferase [ALT], aspartate aminotransferase [AST], alpha-fetoprotein [AFP], and gamma-glutamyl transferase [GGT]), liver stiffness, blood clotting (international normalized ratio and platelets), family history of liver cancer, alcohol use, and diabetes. A few prediction models/scoring systems developed from the general population [8] or patients in the absence of well-established risk factors for liver cancer [9,10] also additionally incorporate lipid profile (total cholesterol [8,9] or triglycerides [10]) and smoking [9,11].

Yet no single scoring system is universally accepted [12]. Moreover, most scoring systems focus on liver cancer of viral etiologies and are only designed for patients with preexisting chronic viral hepatitis [6,7,13]. While a small number of scoring systems concurrently consider liver cancer of other etiologies, these scoring systems are mostly targeted at patients with preexisting liver diseases of different etiologies [6]. Scoring systems developed among the general population or those in the absence of known etiologies remain scarce (Supplementary Table S1) [8,9,11,14]. In addition, even though diabetes is associated with liver cancer [4], scoring systems have rarely been developed among diabetes patients (Supplementary Table S1) [10,15], nor has subgroup analysis on diabetes patients been commonly performed among studies focusing on the general population [8].

While Cox regression is traditionally applied to develop liver cancer prediction models or risk scoring systems, several studies have incorporated machine learning algorithms to complement the standard approach. Tree-structured algorithms are the most commonly applied [8,16,17,18,19] machine learning algorithm due to its ease of interpretation. Few studies also tried artificial neural network [17,20] and support vector machine [17]. Nevertheless, the focus of machine learning application is largely on performance improvement. In a study performed in South Korea, An et al. [8] has applied Cox regression to guide variable selection, and developed a predictive model using random survival forest and extreme gradient boosting models with the predetermined set of variables. However, while some proposed the use of web-based application system to return the results of machine learning-based prediction models [17], the lack of explicit scoring systems of machine learning-based models [8,17] may hinder its clinical application.

On the contrary, Xie et al. [21] has recently proposed an interpretable machine learning framework to develop risk scoring system for time-to-event outcome, where random survival forest (decorrelated trees) is first applied to guide variable selection, and the set of selected variables is subsequently assigned weight using Cox regression to generate a scoring system. The scoring system is subject to fine-tuning based on clinical expert knowledge before finalization. The advantages of the proposed framework are that (i) tree-structured algorithms are intrinsically interpretable; (ii) selection of variables is guided by random survival forest, which may provide a more objective approach to selecting less established risk factors; (iii) random survival forest reduces variance in feature selection in individual trees; (iv) Cox regression remains the most widely accepted approach to developing scoring system for survival data; (v) domain knowledge is incorporated into scoring system development; and (vi) development of a scoring system may enable clinical interpretation and application.

To fill the gaps in the literature on (i) the lack of prediction models for liver cancer among diabetes patients who are at greater risk of developing liver cancer than the general population; and (ii) the lack of prediction models among patients in the absence of liver diseases, this study aims to (i) develop a scoring system for predicting the risk of liver cancer over time among diabetes patients who received care in primary care settings; and (ii) develop a sub-model for diabetes patients in the absence of chronic hepatitis B/C or liver cirrhosis, using a random survival forest-guided approach.

## 2. Methods

### 2.1. Study Design and Study Population

A retrospective cohort study was conducted using territory-wide electronic health records of Hong Kong’s public healthcare system. Under Hong Kong’s dual-track healthcare system, the public sector provides approximately 90% inpatient care and 30% outpatient services (including specialist and primary care levels) to the general public. The Hospital Authority (HA) is a statutory body responsible for managing 43 hospitals, 49 specialist outpatient clinics and 74 general outpatient clinics (GOPCs). Since late 2009, the HA has launched a diabetes mellitus complication screening (DMCS) assessment programme in addition to usual diabetes care at GOPCs.

The HA maintains a centralized clinical data repository to store records on patients’ demographics, inpatient admissions and outpatient attendances, prescription records, disease diagnoses, and laboratory test results. Disease diagnoses were coded according to the International Classification of Disease 9th or 10th revision (ICD-9 or ICD-10), or the International Classification of Primary Care 2nd edition (ICPC-2). Data were accessed via HA Data Collaboration Lab. 

### 2.2. Patients

Patients who were (i) diagnosed with type 2 diabetes mellitus at the age of 18 years or above; and (ii) referred for a first DMCS assessment by a physician at any of the GOPCs between 2010 and 2019 were initially included. Index date was defined as date of the first assessment. Those who had (i) a missing date of diabetes diagnosis; (ii) a history of malignancy; or (iii) less than six months of follow-up were excluded. Patients were followed up until a liver cancer diagnosis, death, or 31 December 2019. To address class imbalance, patients who developed liver cancer (*n* = 1995) and an equal number of random subset of patients who did not develop any cancer during follow-up were selected. Patients with no information on any of the liver function tests examined (ALT, AST, AFP, and GGT) were further excluded (*n* = 26). 

### 2.3. Input Variables

Input variables were ascertained when patients received a first DMCS assessment. Candidate variables for scoring were demographics (sex and age), duration of diabetes, medical history (chronic hepatitis B/C, liver cirrhosis, ischemic heart disease, cerebrovascular disease, heart failure, hypertension, chronic kidney disease, chronic obstructive pulmonary disease, pneumonia, and family history of diabetes), medication use (anti-diabetic drugs: metformin, sulfonylurea, insulin, and dipeptidyl peptidase-4 inhibitors, aspirin, non-steroidal anti-inflammatory drugs, anti-coagulants, anti-platelet drugs, anti-hypertensive drugs [22], and statins), behaviors (alcohol use and smoking), anthropometric measurements (body mass index and waist-to-hip ratio), and laboratory measurements (ALT, glycated hemoglobin, fasting glucose, low-density lipoprotein cholesterol, high-density lipoprotein cholesterol, triglycerides, and serum creatinine). Since three other liver enzymes (AST, AFP, and GGT) were less commonly measured (51, 62, and 50% respectively) among the study cohort, only ALT was included as candidate variable. Medication use was defined as whether patients had been prescribed a drug at the time of the assessment. Laboratory results were taken from measurements closest to the assessment date.

### 2.4. Outcome Variable

The outcome of interest was diagnosis of liver cancer (ICD-9: 155; ICD-10: C22) during follow-up.

### 2.5. Data Analysis

For model building, patients were randomly split into training, validation, and test sets in a 7:1:2 ratio by default. Random survival forest was first applied to rank variable importance. According to the ranking in variable importance, each variable was sequentially added to the initial set of variables in the scoring model until no further model performance (AUC) on validation set was shown. Variables were then selected by considering model performance improvement and model parsimony. Cox proportional hazards regression was subsequently applied for weight assignment. For continuous variables, categories for weight assignment were determined by default quantiles. The scoring system was fine-tuned based on clinical expert knowledge before finalization. The number of trees in the random survival forest was set as 30. Model performance was evaluated using Harrell’s concordance (C−) index and AUC as metrics. Liver cancer-free survival during follow-up across different risk score intervals was examined using Kaplan-Meier method. A sub-model was developed among patients in the absence of chronic hepatitis B/C or liver cirrhosis.

## 3. Results

Of the 384,121 patients identified, 1995 patients developed liver cancer during a median follow-up of 6.2 years. The liver cancer incidence was 0.92 per 1000 person-years. Table 1 shows the baseline characteristics of patients who developed liver cancer and a subset of patients (*n* = 1969) who remained cancer-free during follow-up. Those who developed liver cancer were more likely to be older (66.90 vs. 62.17 years, *p* < 0.001), male (74.24 vs. 49.42%, *p* < 0.001), have liver cirrhosis (13.38 vs. 2.23%, *p* < 0.001), chronic hepatitis B (14.39 vs. 1.12%, *p* < 0.001), and a higher ALT (46.17 vs. 27.48 U/L, *p* < 0.001). Of the patients selected, 1527 patients who developed liver cancer and 1907 patients who remained cancer-free during follow-up did not have a history of liver cirrhosis or chronic hepatitis B/C.

### 3.1. Final Scoring System (Score_final_)

Chronic hepatitis B/C status, ALT, age, liver cirrhosis status, and sex were ranked as top five variables contributing to the risk of liver cancer in descending order.

In the final 100-point scoring system, presence of chronic hepatitis B/C (maximum 14 points), elevated ALT (maximum 25 points), older age (maximum 41 points), presence of liver cirrhosis (8 points), and being male (12 points) were assigned positive weights contributing to an increased risk of liver cancer development. Patients from the age of 47 years onwards were assigned positive weights accounting for elevated liver cancer risk due to chronological aging. Also, presence of chronic hepatitis B alone was assigned slightly greater weights towards liver cancer development than presence of chronic hepatitis C alone or coinfection of chronic hepatitis B/C. In addition, ALT started to carry positive weights at the level of 16.2 U/L or above, and the weights rose markedly from 47 U/L onwards (Table 2).

### 3.2. Liver Cancer Development during Follow-up

**Figure 1:**
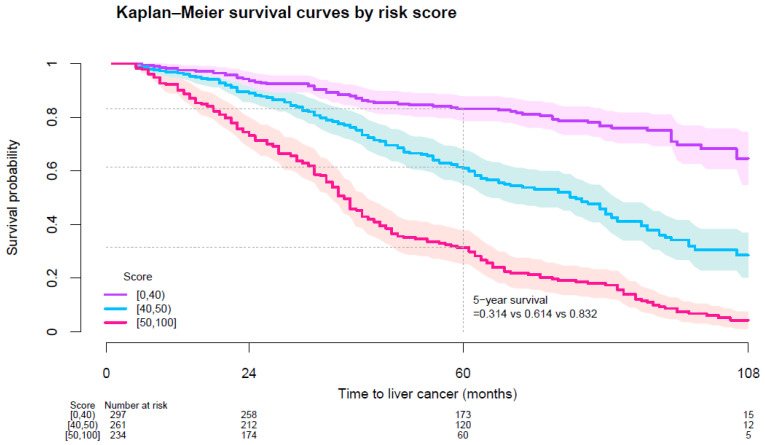
Kaplan-Meier liver cancer-free survival curves among diabetes patients on test set by risk score.

At 5 years of follow-up, the liver cancer-free survival probability among patients on test set with scores 0 to 39, 40 to 49, and 50 or above were 0.832, 0.614, and 0.314 respectively (Supplementary Table S2; Figure 1). Median time to liver cancer development among patients with scores 40 to 49 and 50 or above were 6.67 and 3.42 years respectively (Figure 1). 

### 3.3. Absence of Chronic Hepatitis B/C or Liver Cirrhosis (Score_no liver disease_)

In the absence of chronic hepatitis B/C or liver cirrhosis, ALT, age, triglycerides, and sex were identified as top four important variables contributing to the risk of liver cancer in descending order. Weight assignment to ALT, age, and sex among patients in the absence of liver diseases was consistent with that among the overall diabetes population (Table 2 and Table 3). However, triglycerides level was found to be inversely associated with the risk of liver cancer (Table 3).

### 3.4. Model Performance

The C-indexes of Score_final_ and Score_no liver disease_ on test sets were 0.706 (95%CI: 0.676–0.741) and 0.704 (95%CI: 0.677–0.739) respectively. The corresponding 5-year AUCs were 0.739 (95%CI: 0.694–0.784) and 0.735 (95%CI: 0.701–0.789).

## 4. Discussion

The current study applied random survival forest to extract information derived from multidimensional relationships among covariates in individual survival trees, and convert the aggregate information into a parsimonious risk score for liver cancer prediction targeted at diabetes patients encountered in primary care settings in the presence or absence of liver diseases (chronic hepatitis B/C or liver cirrhosis). Consistent with the literature, elevated ALT is a strong risk factor for liver cancer regardless of liver disease status. While most of the existing models focus on patients with preexisting liver diseases, this study additionally developed a sub-model among diabetes patients in the absence of liver diseases, where triglycerides level was found to be a potential predictor of liver cancer.

Consistent with the existing risk prediction models [13,23,24,25,26], the proposed scoring system incorporated elevated ALT as risk factor for liver cancer among diabetes patients. Compared to other risk prediction models for liver cancer among diabetes patients or the general population, ALT was the most commonly used liver function indicator [8,9,11,14,15]. In a risk model developed among diabetes patients [10], GGT was also included as predictor. In the existing models among the general population, ALT at a level ranging from 20 to 30 U/L [9,11,14] was incorporated as risk factor for liver cancer. In another model among diabetes patients [15], the threshold of ALT for elevated risk of liver cancer was 45 U/L above. The proposed scoring system in the current study identified ALT ≥ 16.2 U/L indicating a mildly increased risk of liver cancer and ALT ≥ 47 U/L indicating a markedly elevated risk of liver cancer. The optimal cutoffs determined by the algorithm were comparable to those in the literature.

The proposed system also identified that serum triglycerides is an important variable associated with the risk of liver cancer, and triglycerides level is inversely associated with the risk of liver cancer among diabetes patients in the absence of chronic viral hepatitis or liver cirrhosis. This is consistent with the existing prediction models for liver cancer among the general population [8,9] or diabetes patients [10]. While elevated triglycerides or elevated total cholesterol is known for their associations with an increased risk of cardiovascular events, in two Korean risk prediction models [8,9] for liver cancer among the general population, total cholesterol was found to be negatively linked to the risk of liver cancer. In another liver cancer risk prediction model developed among diabetes patients in Korea [10], only age, triglycerides, and GGT were included as predictors in the final model, where triglycerides below 1.69 mmol/L was a risk factor for liver cancer. In the sub-model among patients in the absence of liver diseases in the current study (Score_no liver disease_), triglycerides below 1.94 mmol/L indicates an elevated risk of liver cancer. The actual threshold of triglycerides for elevated liver risk falls within 0.84 and 1.94 mmol/L, which is consistent with the findings of previous research [10]. The associations between serum lipids and liver cancer risk remain controversial in epidemiological studies [27,28], and the plausible mechanisms linking the possible inverse relationships between triglycerides/total cholesterol and liver cancer remain largely unknown. Possible mechanisms include impaired liver function leading to decreased cholesterol synthesis [27], high demand for lipid uptake from underlying liver tumor cells [27,29], and compromised immune function due to low lipid levels [27,30,31].

The final scoring system implies that older age, male sex, presence of chronic hepatitis B/C, liver cirrhosis, or elevated ALT (≥47 U/L) could be individually and collectively linked to an increased risk of liver cancer development. Furthermore, consistent with previous studies [10], the sub-model (Score_no liver disease_) suggests that a decreased level of triglycerides could be associated with a higher risk of liver cancer.

There are several limitations in the current study. First, this study only considered ALT as input variable since other liver function tests were less commonly performed among the study cohort. Second, external validation of the proposed scoring system was not available in this study. However, internal validation was performed on the unseen test set. Third, this study was based on a large database and may lack the level of granularity to address certain clinical research questions. Lastly, generalizability of the findings could be limited to Asian diabetes population. Future research is warranted to evaluate the effectiveness of the proposed scoring system in practical applications, and explore the applicability across different populations. Another future research direction is to explore a broader list of factors potentially associated with the risk of liver cancer incidence in order to improve performance and comprehensiveness of the models.

## 5. Conclusions

The current study developed a scoring system for predicting the risk of liver cancer over time among diabetes patients. According to age, sex, chronic hepatitis B/C status, liver cirrhosis, and ALT, the relative trajectories of liver cancer development can be estimated for risk stratification. Also, in the absence of cirrhosis or chronic hepatitis B/C, triglycerides level could be a potential predictor of liver cancer.

## Figures and Tables

**Table 1:** Baseline characteristics of diabetes patients who developed liver cancer and those who remained free of cancer during follow-up.

	Liver Cancer		No Liver Cancer	
Characteristics	(*n* = 1995)		(*n* = 1969)	
Demographics				
Male, *n* (%)	1481 (74.24%)		973 (49.42%)	
Age at assessment in year, mean ± SD	66.90 ± 9.92		62.17 ± 11.80	
Duration of diabetes in year, median (IQR)	6 (1–11)		3 (1–9)	
Medical history				
Chronic hepatitis status				
Chronic hepatitis B infection only, *n* (%)	287 (14.39%)		22 (1.12%)	
Chronic hepatitis C infection only, *n* (%)	62 (3.11%)		2 (0.10%)	
Coinfection of chronic hepatitis B and C, *n* (%)	3 (0.15%)		0 (0%)	
Liver cirrhosis, *n* (%)	267 (13.38%)		44 (2.23%)	
Ischemic heart disease, *n* (%)	163 (8.17%)		137 (6.96%)	
Cerebrovascular disease, *n* (%)	140 (7.02%)		127 (6.45%)	
Heart failure, *n* (%)	65 (3.26%)		34 (1.73%)	
Hypertension, *n* (%)	1765 (88.47%)		1695 (86.08%)	
Chronic kidney disease, *n* (%)	185 (9.27%)		317 (16.10%)	
Chronic obstructive pulmonary disease, *n* (%)	32 (1.60%)		9 (0.46%)	
Pneumonia, *n* (%)	104 (5.21%)		52 (2.64%)	
Family history of diabetes, *n* (%)	762 (38.20%)		931 (47.28%)	
Medication use				
Anti-diabetic drugs				
Metformin, *n* (%)	1066 (53.43%)		779 (39.56%)	
Sulfonylurea, *n* (%)	862 (43.21%)		529 (26.87%)	
Insulin, *n* (%)	221 (11.08%)		112 (5.69%)	
Dipeptidyl peptidase-4 inhibitors, *n* (%)	69 (3.46%)		71 (3.61%)	
Glucosidase inhibitors, *n* (%)	13 (0.65%)		11 (0.56%)	
Meglitinide, *n* (%)	1 (0.05%)		0 (0%)	
Glitazone, *n* (%)	10 (0.50%)		4 (0.20%)	
Sodium-glucose cotransporter-2 inhibitors, *n* (%)	1 (0.05%)		6 (0.30%)	
Glucagon-like peptide-1 receptor agonists, *n* (%)	1 (0.05%)		0 (0%)	
Any of the above, *n* (%)	1395 (69.92%)		1000 (50.79%)	
Aspirin, *n* (%)	457 (22.91%)		406 (20.62%)	
Non-steroidal anti-inflammatory drugs, *n* (%)	931 (46.67%)		1076 (54.65%)	
Anti-coagulants, *n* (%)	96 (4.81%)		103 (5.23%)	
Anti-platelet drugs, *n* (%)	77 (3.86%)		131 (6.65%)	
Anti-hypertensive drugs, *n* (%)	1446 (72.48%)		1363 (69.22%)	
Statins, *n* (%)	594 (29.77%)		1011 (51.35%)	
Behaviors				
Current drinker/ex-drinker, *n* (%)	807 (40.45%)		562 (28.54%)	
Current smoker/ex-smoker, *n* (%)	991 (49.67%)		547 (27.78%)	
Anthropometric measurements				
Body mass index in kg/m^2^, mean ± SD	25.90 ± 4.05		26.19 ± 4.29	
Waist-to-hip ratio, mean ± SD	0.96 ± 0.07		0.93 ± 0.06	
Laboratory measurements				
Alanine transaminase in U/L, mean ± SD	46.17 ± 44.76		27.48 ± 52.57	
HbA_1c_ in %, mean ± SD	7.42 ± 1.52		7.44 ± 1.50	
Fasting glucose in mmol/L, mean ± SD	7.65 ± 2.31		7.66 ± 2.25	
Low-density lipoprotein cholesterol in mmol/L, mean ± SD	2.53 ± 0.76		2.69 ± 0.80	
High-density lipoprotein cholesterol in mmol/L, mean ± SD	1.30 ± 0.39		1.26 ± 0.33	
Triglycerides in mmol/L, mean ± SD	1.33 ± 0.92		1.65 ± 1.28	
Serum creatinine in µmol/L, mean ± SD	88.69 ± 46.66		79.92 ± 32.03	

Abbreviations: HbA_1c_, glycated hemoglobin; IQR, interquartile range; SD, standard deviation.

**Table 2:** Liver cancer risk scoring system among diabetes patients (Score_final_).

Variable	Value	Point
Chronic hepatitis B/C	None	0
	Chronic hepatitis B-only	14
	Chronic hepatitis C-only	10
	Coinfection of chronic hepatitis B and C	10
Alanine aminotransferase, U/L	<16.2	0
	[16.2, 47)	10
	[47, 96)	20
	≥96	25
Age, years	<47	0
	[47, 55)	15
	[55, 75)	26
	[75, 83)	34
	≥83	41
Liver cirrhosis	Absent	0
	Present	8
Sex	Female	0
	Male	12

**Table 3:** Liver cancer risk scoring system among diabetes patients in the absence of liver cirrhosis or chronic viral hepatitis B/C (Score_no liver disease_).

Variable	Value	Point
Alanine aminotransferase, U/L	<10.9	1
	[10.9, 16)	0
	[16, 44)	12
	[44, 85.9)	25
	≥85.9	31
Age, years	<47	0
	[47, 55)	22
	[55, 75)	34
	[75, 83)	43
	≥83	46
Triglycerides, mmol/L	<0.6	10
	[0.6, 0.84)	9
	[0.84, 1.94)	6
	[1.94, 3.14)	0
	≥3.14	1
Sex	Female	0
	Male	13

## Data Availability

Data is not available for sharing due to access restriction.

## Supplementary Materials

The following supporting information can be downloaded at: https://www.mdpi.com/article/10.3390/cancers16132310/s1, Table S1: Summary of selected liver cancer risk prediction or scoring systems among the diabetes or general population; Table S2: Liver cancer-free survival probability among diabetes patients on test set by score interval (Score_final_) at different follow-up time points.
