# Supplementary material for: Scoring System for Predicting the Risk of Liver Cancer among Diabetes Patients: A Random Survival Forest-Guided Approach
Source: Cancers (Basel). 2024 Jun 24;16(13):2310. doi: 10.3390/cancers16132310 (PMC11240698; doi:10.3390/cancers16132310)
Supplement: Supplementary file 1 [file cancers-16-02310-s001.zip › cancers-3019032-supplementary.pdf]

Table S1. Summary of selected liver cancer risk prediction or scoring systems among the diabetes or general population.

	Region	Study population	Follow-up	Sample size (Number of liver cancer cases/ Total number of patients)	Scoring system	Variable selection	Prediction model/ scoring assignment	Number of parameters	Liver cancer outcome
Diabetes population									
Si et al. (2016)	South Korea	Aged ≥18 years, Absence of chronic viral hepatitis/ alcoholic cirrhosis	Median: 55 months	36/3544	Yes	Cox regression	Logistic regression	3	10-year risk
Rau et al. (2016)	Taiwan	Aged 21-90 years	NA	515/2060	No	Chi-square test	Artificial neural network; logistic regression	10	6-year risk
Li et al. (2018)	Taiwan	Aged 30-84 years	Mean: 8.33 years	748/31723	Yes	Cox regression	Cox regression	11	3-, 5-, 10-year risk
General population									
Michikawa et al. (2012)	Japan	Aged 40-69 years	Mean: 12.6 years	104/17654	Yes	Cox regression	Cox regression	8	10-year risk
Wen et al. (2012)	Taiwan	Aged ≥20 years	Mean: 8.5 years	1668/428584	Yes	Existing literature	Cox regression	4-10	5-, 10-year risk
Sinn et al. (2020)	South Korea	Aged ≥20 years, Absence of chronic viral hepatitis/ heavy alcohol use/ cirrhosis	Median: 8.0 years	236/467206	Yes	Cox regression	Cox regression	6	5-, 10-year risk
An et al. (2021)	South Korea	Aged 40-80 years	Median: 11.1 years	2189/417346	No	Cox regression	Cox regression; random survival forest; extreme gradient boosting	14	9-year risk; time to liver cancer

NA, not applicable.

Table S1. Summary of selected liver cancer risk prediction or scoring systems among the diabetes or general population (continued).

	Demographics			Liver diseases										
	Age	Sex	Income	Cirrhosis	Alcoholic cirrhosis	Other cirrhosis	Chronic hepatitis B/C	Alcoholic hepatitis	Viral hepatitis	Other chronic hepatitis	Alcoholic fatty liver disease	Other fatty liver disease	Chronic liver disease	Family history of chronic liver disease
Diabetes population														
Si et al. (2016)	X													
Rau et al. (2016)	X	X			X	X		X	X	X	X	X		
Li et al. (2018)	X	X		X			X							
General population														
Michikawa et al. (2012)	X	X					X							
Wen et al. (2012)	X	X					X							
Sinn et al. (2020)	X	X												
An et al. (2021)	X	X	X				X						X	X

Table S1. Summary of selected liver cancer risk prediction or scoring systems among the diabetes or general population (continued).

	Behavioral factors				Obesity	Diabetes-related factors		Lipid profile				
	**Smoking**	**Alcohol use**	**Physical Activity**	**Coffee consumption**	**BMI**	**Diabetes**	**Variation in HbA_{1c}**	**THR**	**Total cholesterol**	**Triglyceride**	**Dyslipidemia**	**Hyperlipidemia**
Diabetes population												
Si et al. (2016)										X		
Rau et al. (2016)												X
Li et al. (2018)	X						X	X				
General population												
Michikawa et al. (2012)		X		X	X	X						
Wen et al. (2012)	X	X	X			X						
Sinn et al. (2020)	X					X			X			
An et al. (2021)					X	X			X		X	

BMI, body mass index; HbA_{1c}, glycated hemoglobin; THR, total cholesterol to high-density lipoprotein cholesterol ratio.

Table S1. Summary of selected liver cancer risk prediction or scoring systems among the diabetes or general population (continued).

	Medication use		Liver function				Other diseases	
	**Anti-diabetic drugs**	**Lipid-lowering drugs**	**ALT**	**AST**	**AFP**	**GGT**	**HIV infection**	**Mental disorder**
Diabetes population								
Si et al. (2016)						X		
Rau et al. (2016)								
Li et al. (2018)	X	X	X					
General population								
Michikawa et al. (2012)			X					
Wen et al. (2012)			X	X	X			
Sinn et al. (2020)			X					
An et al. (2021)			X			X	X	X

AFP, alpha fetoprotein; ALT, alanine aminotransferase; AST, aspartate transaminase; GGT, gamma-glutamyl transferase.

Table S2. Liver cancer-free survival probability among diabetes patients on test set by score interval ($Score_{final}$) at different follow-up time points.

	**Score interval**		
**Time (t)**	**[0, 40)**	**[40, 50)**	**[50, 100]**
t=2 years	0.943	0.895	0.745
t=5 years	0.832	0.614	0.314
t=7 years	0.768	0.459	0.180

References

1. Si WK, Chung JW, Cho J, et al. Predictors of increased risk of hepatocellular carcinoma in patients with type 2 diabetes. *PLoS One*. 2016;11(6):e0158066.
2. Rau HH, Hsu CY, Lin YA, et al. Development of a web-based liver cancer prediction model for type II diabetes patients by using an artificial neural network. *Comput Methods Programs Biomed*. 2016;125:58-65.
3. Li TC, Li CI, Liu CS, et al. Risk score system for the prediction of hepatocellular carcinoma in patients with type 2 diabetes: Taiwan Diabetes Study. *Semin Oncol*. 2018;45(5-6):264-74.
4. Michikawa T, Inoue M, Sawada N, et al. Development of a prediction model for 10-year risk of hepatocellular carcinoma in middle-aged Japanese: the Japan Public Health Center-based Prospective Study Cohort II. *Prev Med*. 2012;55(2):137-43.
5. Wen CP, Lin J, Yang YC, et al. Hepatocellular carcinoma risk prediction model for the general population: the predictive power of transaminases. *J Natl Cancer Inst*. 2012;104(20):1599-611.
6. Sinn DH, Kang D, Cho SJ, et al. Risk of hepatocellular carcinoma in individuals without traditional risk factors: development and validation of a novel risk score. *Int J Epidemiol*. 2020;49(5):1562-71.
7. An C, Choi JW, Lee HS, et al. Prediction of the risk of developing hepatocellular carcinoma in health screening examinees: a Korean cohort study. *BMC Cancer*. 2021;21(1):755.
